# Quasi-Static Shear Test of Hybrid Adhesive Bonds Based on Treated Cotton-Epoxy Resin Layer

**DOI:** 10.3390/polym12122945

**Published:** 2020-12-09

**Authors:** Martin Tichý, Viktor Kolář, Miroslav Müller, Rajesh Kumar Mishra, Vladimír Šleger, Monika Hromasová

**Affiliations:** 1 Department of Material Science and Manufacturing Technology, Faculty of Engineering, Czech University of Life Sciences Prague, Kamycka 129, 165 00 Prague, Czech Republic; martintichy@tf.czu.cz (M.T.); vkolar@tf.czu.cz (V.K.); muller@tf.czu.cz (M.M.); 2 Department of Mechanical Engineering, Faculty of Engineering, Czech University of Life Sciences Prague, Kamycka 129, 165 00 Prague, Czech Republic; sleger@tf.czu.cz; 3 Department of Electrical Engineering and Automation, Faculty of Engineering, Czech University of Life Sciences Prague, Kamycka 129, 165 00 Prague, Czech Republic; hromasova@tf.czu.cz

**Keywords:** mechanical behavior, fatigue and fracture mechanics, green composite, biological fabric, alkali treatment, SEM analyses

## Abstract

This research evaluates the mechanical properties of hybrid adhesive bonds with various 100% cotton fabrics in static and quasi-static conditions and the influence of alkali surface treatment (NaOH) of the cotton fabrics on the mechanical properties. Biological fibers in polymers are characterized by low wettability with the matrix, which decreases mechanical properties. Adhesive bonds usually operate in cyclic stress, which causes irreversible failure before maximal strength. In this paper, a quasi-static test was used to load the adhesive bonds in 5–50% (192–1951 N) and 5–70% (192–2732 N) intervals with 1000 cycles. The results of SEM analysis showed good wettability of alkali treated cotton fabric with NaOH solution in hybrid adhesive bonds. The static test proved the influence of reinforcing cotton fabrics on shear tensile strength against pure resin, i.e., sample Erik up to 19% on 14.90 ± 1.15 MPa and sample Tera up to 21% on 15.28 ± 1.05 MPa. The adhesive bonds with pure resin did not resist either quasi-static tests. Reinforcing cotton fabrics resisted both quasi-static tests, even shear tensile strength increases up to 10% on 16.34 ± 1.24 MPa for the fabric Erik. The results of strain difference of adhesive bonds with Tera and Erik confirmed that a lower value of the difference during cyclic loading positively influenced the ultimate shear tensile strength.

## 1. Introduction

Adhesive bonding technology currently presents one of the prospective methods of bonding various materials. The bonding of various materials has applications in, for example, automotive, aircraft and electrotechnics industries [1,2]. A dynamic progression of the adhesive technology is given by various options that this process offers compared to conventional bonding options (welding, soldering, etc.). The significant advantages of adhesive bonding are the wide spectrum of bonded materials, low cost and energy consumption. The adhesive bonding technology can also play supportive roles, such as sealing, clamping and fixing [3,4]. Today, several research institutes deal with adhesive bonds research. Most of them deal with the strength of adhesive bonds [5,6,7]. Factors that influence the strength of adhesive bonds are physical and chemical factors (wettability, adhesion and cohesion) [3], technology factors (roughness and structure surface) [8,9] and construction factors (design, dimension and loading type) [10,11].

Structural epoxy resins are favorite types of commercial adhesives, and they are suitable for bonding technology [12,13]. This is given by their good mechanical properties, chemical resistance, electric resistance, etc. Due to a wide range in selection of epoxy resins, a product that is most suitable for operating conditions can be obtained [14,15]. Therefore, epoxy resins are used in many industries from aircraft construction to epoxy coating production [14,16,17]. Significant changes in properties of an epoxy resin can be achieved by adding a filler, with respect to reinforcement [18,19,20].

A synergy effect, i.e., effect of common action between matrix and reinforcement, leads to mechanical characteristic improvement. The effect is commonly known in particle reinforcement [21,22,23]. A general problem is irregular distribution of particles in a matrix, e.g., sedimentation in the adhesive layer [24]. Synthetic and biologic fibers are appropriate alternatives [24,25]. Specific properties of fibers, such as materials, the orientation of fibers, mass and wettability with the matrix, are basic factors that influence their utilization [26,27,28]. The utilization of glass fabric in adhesive bonds proved to significantly influence shear tensile strength [29]. A current trend in polymer composites is heading to biological material utilization. Bio-composites can be formed [30,31,32,33], and bio-composites are an alternative for research in biologic fiber applications [34].

Biological fibers from cotton, sisal, flax and jute are the most often used cellulose based fibers in various industrial fields [35,36,37]. These fibers are an appropriate (low cost) alternative to synthetic fibers [38,39]. Biological fibers are used mainly in low loading parts, i.e., in car interiors and other vehicles. Such fibers are used mainly in fabric, thread and mat forms [40].

Biological fibers in polymer composite are characterized by low wettability with the matrix, which decreases the mechanical properties of the composite, while the fibers themselves have relatively good mechanical properties [41,42]. Low wettability of biological fibers is given by undesirable surfaces, i.e., hemicellulose residues, lignin, pectin and oils. These components have many hydroxyl groups and give a hydrophobic character to fibbers. Hydrophobic fibers with a hydrophobic matrix (resin) create low wettability, which leads to the negative influence of mechanical properties of polymer composites [43,44]. Improvement of the wettability with respect to the fiber surface structure can be reached by alkaline treatment with NaOH solution [37,45,46,47,48,49]. The improvement of a fiber surface structure was confirmed in a polylactic acid (PLA)/hemp model system [50]. The alkaline surface treatment leads to the removal of lignin, hemicellulose, wax and oils and the depolymerization of cellulose [51,52]. A result of the treatment is an increase in fiber roughness [43,53].

Operating conditions of adhesive bonds usually cause cyclic stress, i.e., cyclic fatigue. Cyclic fatigue causes irreversible adhesive bond failure before maximal strength. The process itself influences structural bonds at relatively low value of stress, with delamination of the adhesive layer and bonded material, which negatively influences the service life of bonds [54]. The strength and service life of adhesive bonds are decreased even by low stress values with cyclic degradation. Cyclic (quasi-static) tests are necessary for practical application of adhesive bonds [30,54,55,56,57,58].

The aim of this research was to evaluate mechanical properties and service life of hybrid adhesive bonds with various 100% cotton fabrics applied as reinforcement in various cyclic stress conditions. The second aim was to determine the influence of alkali surface treatment of cotton fabric on the mechanical properties of hybrid adhesive bonds.

## 2. Materials and Methods

### 2.1. Materials

The bonded layer of hybrid adhesive bonds was composed from various 100% cotton fabrics and one type of epoxy resin. These two components, as matrix and filler, respectively, created the composite layer. Hybrid adhesive bonds were bonded by the composite layer according to ČSN EN 1465. The mechanical properties of hybrid adhesive bonds based on a cotton–epoxy bonded layer in cyclic shear conditions was the subject of the study. Adhesive bonds were tested by static and quasi-static shear tests to evaluate the mechanical properties (tensile shear strength, strain) and to establish the service life of adhesive bonds in real conditions. 

**Figure 1:**
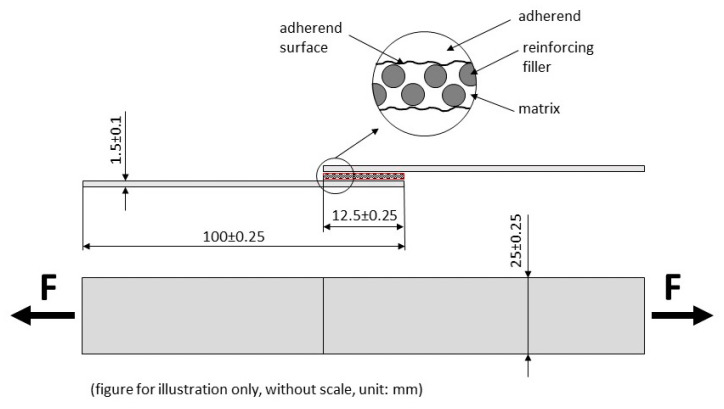
Scheme of hybrid adhesive bond with composite layer.

The composite layer thickness was measured by the Gwyddion program (Czech Metrology Institute, Romana Havelky 294, Jihlava) and SEM images; values are evident in Table 1. The Gwyddion program operates with uploaded SEM images, where individual dimensions of the hybrid adhesive bonds are measured. It is a modular program for data visualization and analysis. It is used for general image processing. Figure 1 shows the scheme of adhesive bonds with dimensions and bond components as adherend and composite layer.

The adherend used for hybrid adhesive bonds was structural carbon steel S235J0 (Ferona a.s., Prague, Czech Republic) with a size of 100 × 25 × 1.5 mm^3^, according to standard ČSN EN 1465. The bonded surface of the adherend was mechanically treated by abrasive Brow Corundum in a blasting chamber and chemically cleaned in an acetone bath. Roughness of the treated adherend surface was Ra = 3.68 ± 0.14 μm and Rz = 11.23 ± 0.45 μm and was measured with a Mitutoyo Surftest 301 (Mitutoyo Europe GmbH, Neuss, Germany) profilometer. 

The matrix was CHS-Epoxy 324 structural epoxy resin (Epoxy 1200) with P11 hardener (Havel Composites CZ s.r.o., Svésedlice, Czech Republic) in a ratio of 100:7 according to a material list of the resin. This epoxy resin is commonly used as a metal bonding adhesive. The fillers of the composite layer were various 100% cotton fabrics (Table 1). The fabrics selected for reinforcement purposes were made from 100% grey cotton. No finishing treatments were given. Grey fabric samples were thoroughly washed for removal of residual starch and pre-weaving surface treatments. They were treated with 5% NaOH solution (Sigma Aldrich-Merck KGaA, Darmstadt, Germany), for 15 min. As is well known, NaOH is a scouring agent used for cotton fabrics in order to remove the oils and fats from the fiber surface. This treatment helps in removal of the impermeable coatings on the fiber surface so that it is ready for bonding with any adhesive material. The chemical treatment of biological reinforcing material is necessary to improve mechanical properties by adhesive strength [44,46,59]. This effect leads to improved interfacial bonding, i.e., adhesive bonds inside composite layer [60,61,62,63].

These two groups of treated and non-treated fabric samples were used for static tests in order to evaluate the influence of treatment technology. Only the treated group of fabrics was used for quasi-static tests.

### 2.2. Methods

The treated and non-treated cotton fabric groups of hybrid adhesive bonds were tested by the static shear tensile test. The static shear tensile test was used to evaluate the influence of treatment technology on mechanical properties of hybrid adhesive bonds. The static shear tensile test measured adhesive bonds at loading speed of 0.6 mm·min^−1^ with 8 samples, according to ČSN EN 1465. 

After static tests, the quasi-static tests followed. The treated cotton group of adhesive bonds was only used for quasi-static tests. The tested group contained 8 samples, and when only one sample did not pass the quasi-static test, all tested groups were considered as insufficient. The method of quasi-static tests was based on shear tensile strength from the static tensile test of pure epoxy resin adhesive bond as a reference value. This static reference value was 3902 N (average maximal force). The quasi-static test loaded the adhesive bonds in 1000 loading cycles with test speed of 6 mm·min^−1^. The quasi-static test included two different loading intervals, i.e., lower loading interval 5–50% (192–1951 N) and higher loading interval 5–70% (192–2732 N) from the static reference value. The time delay on the peak loading interval was 0.5 s. The quasi-static test was finished when the 1000 cycles passed, and the adhesive bond was statically loaded until break with a loading speed of 0.6 mm·min^−1^. The quasi-static test was stopped when destruction occurred before 1000 cycles. Mechanical properties of hybrid adhesive bonds were tested on the universal testing machine LABTest 5.50 ST with measuring unit AST KAF 50 kN and evaluation software Test and Motion (LABORTECH s.r.o., Opava, Czech Republic). The tests were measured under controlled laboratory temperature and humidity.

The composite layer of the hybrid adhesive bond was analyzed by a MIRA 3 TESCAN GMX SE (Tescan Brno s.r.o., Brno, Czech Republic) scanning electron microscope (SEM), i.e., an interaction between reinforcing fabric, matrix and bonded material. SEM analyses was used to show differences between non-treated and treated fabric by 5% NaOH solution. Samples surfaces were gold dusted by a Quorum Q150R ES (Tescan Brno s.r.o., Brno, Czech Republic) device for SEM analysis.

Measured values were evaluated by analysis of variance, i.e., ANOVA F-test using the STATISTICA program. The statistical hypothesis was evaluated at a significance level of 0.05 between etalon and individual variants of the experiment. The statistical hypothesis H_0_ presented statistically nonsignificant differences between measured values (*p* > 0.05). The hypothesis H_1_ rejected the hypothesis H_0_ and presented statistically significant differences between measured values (*p* < 0.05).

## 3. Results

The hybrid adhesive bonds were tested for mechanical properties, i.e., shear tensile strength and strain. The results of tests were split into three parts, which were SEM analyses, static tests and quasi-static tests, where various factors were evaluated. The first part described the structure of the composite layer and the treated fabrics. The second part determined if the fabrics had an influence on the static mechanical properties of the hybrid adhesive bonds against pure epoxy resin. Furthermore, the influence of 5% NaOH treatment on the static mechanical properties of treated fabric as compared to non-treated fabric was studied. The third part followed the mechanical properties of hybrid adhesive bonds using quasi-static tests and compared them with the results of static tests. In addition, a loading curve of quasi-static test was evaluated. 

**Figure 2:**
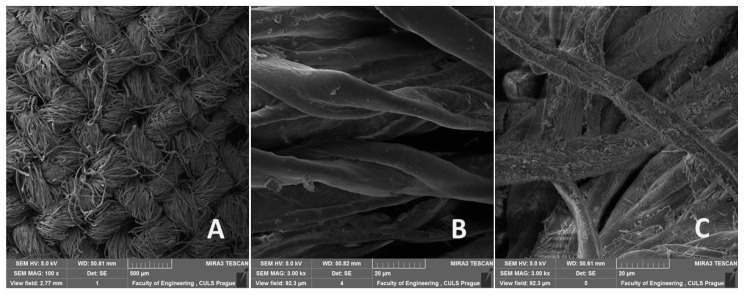
SEM images of cotton reinforcing fabric Bjaz (HV 5.0 kV). (**A**) Overview of the reinforcing fabric (mag 100×, view field 2.77 mm, WD 50.81 mm), (**B**) surface structure of cotton fibers non-treated (3. 00 kx, view field 92.3 µm, WD 50.82 mm), (**C**) surface structure of cotton fibers chemically treated by 5% NaOH solution (mag 3.00 kx, view field 92.3 µm, WD 50.61 mm).

SEM analysis described the structure of the composite layer and treated fabrics. The results of electron microscopy showed significant differences in the tested fabric surface between treated and non-treated by 5% NaOH solution. Figure 2 shows a view of the reinforcing fabric for the composite layer. Figure 2B,C show longitudinal images of the fiber surface before and after treatment with 5% NaOH solution. Interestingly, the alkali treatment generated micro crystalline compounds of cellulose adhering to the fiber surface. These micro-crystalline compounds provided additional bonding sites and enhanced the interface for the adhesive layer of the resin. Such crystals acted as a third phase in the composite, and the increased interfacial surface provided by the crystals enhanced the mechanical performance in the composites.

**Figure 3:**
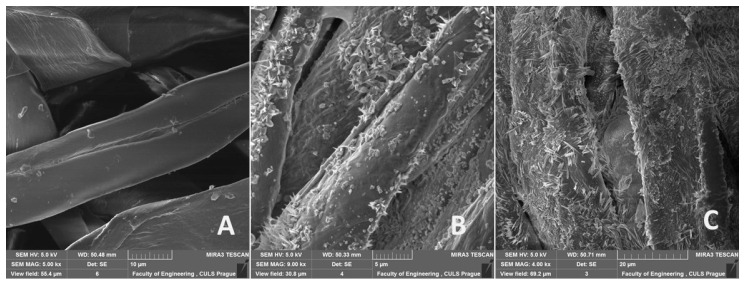
SEM images of cotton reinforcing fabric (HV 5.0 kV). (**A**) Surface structure of cotton fibers non-treated—fabric Thomas Alan (mag 5.00 kx, view field 55.4 µm, WD 50.48 mm), (**B**) surface structure of cotton fibers chemically treated by 5% NaOH solution—fabric Thomas Alan (mag 9.00 kx, view field 30.8 µm, WD 50.33 mm), (**C**) surface structure of cotton fibers chemically treated by 5% NaOH solution—fabric Bjaz (4.00 kx, view field 69.2 µm, WD 50.71 mm).

It can be seen that the density of the micro-crystalline particles is higher in Figure 3C (Bjaz) as compared to Figure 3B (Thomas). As the sample 1 (Bjaz) was lighter in construction, it provided easier penetration of alkali solution into the interstices of yarns and fibers. Thus, there was more abundance of the micro particles. On the other hand, fabric Thomas was relatively heavier and was woven with a twill construction. Typically, in a twill fabric, there is higher cohesiveness of yarn floats (un-interlaced segments), which cause a jamming condition. Such fabrics are less porous as compared to canvas or plain geometry in woven fabrics. Twill constructions do not allow infusion of alkali as effectively as plain/canvas construction. This may be the reason for the relatively lower amount of crystals visible on the fiber surface. 

**Figure 4:**
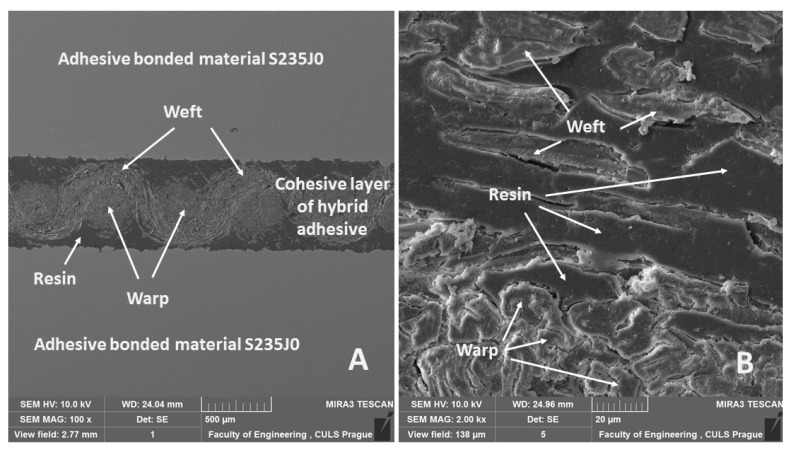
SEM images of hybrid adhesive bond cross-section (HV 10.0 kV). (**A**) Adhesive bond disposition (mag 100×, view field 5.7 mm, WD 24.04 mm), (**B**) detailed view of adhesive bond layer (mag 2.00 kx, view field 138 µm, WD 24.96 mm).

Scanning electron microscopy (SEM) was used to evaluate the quality of the adhesive bonds, i.e., interaction of adhesion and cohesion forces and possible initiation of cracks at cyclic loading. Figure 4A shows a cross-section of the hybrid adhesive bond, which consisted of bonded material and composite layers. A disposition of hybrid adhesive bond and an interface is evident in Figure 4A. The composite layer consists of fabric, which is split to load warp and weft elements. The sandblasting texture of bonded material is evident in Figure 4A. The results showed a low adhesive bond between resin, warp and weft in some place, i.e., the reinforcing fabric was not optimally wetted/impregnated in some places, which may have generated cracks during mechanical testing. 

**Figure 5:**
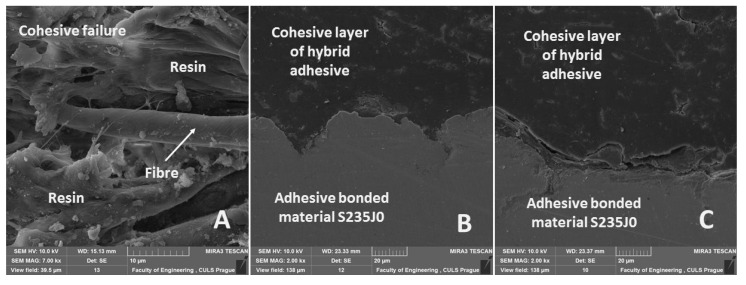
SEM images of hybrid adhesive bond—fabric Bjaz (HV 10.0 kV). (**A**) Fracture surface of hybrid adhesive bond after destruction (mag 7.00 kx, view field 39.5 µm, WD 15.13 mm), (**B**) cross-section of non-loaded adhesive bond (mag 2.00 kx, view field 138 µm, WD 23.33 mm), (**C**) cross-section of cyclically loaded adhesive bond (mag 2.00 kx, view field 138 µm, WD 23.37 mm).

SEM analysis of the fracture surface showed good wettability of chemically treated fabrics, i.e., good interaction with resin (Figure 5A). A cross-section of the non-loaded adhesive bond is evident in Figure 5B, where good wettability and good adhesive interactions are evident. A cross-section of loaded adhesive bond after 1000 loading cycles without a subsequent break is evident in Figure 5C. Figure 5C shows a fracture initiation at the interface between the composite layer and the bonded material, which led to adhesive failure.

A more significant application of natural fibers in composites is hindered by their low adhesion to a polymer matrix, i.e., low adhesive forces between biological fiber and resin. This deficit can be eliminated by alkali treatment of the fiber, which was confirmed by the results [62].

The results of the static test confirmed the significant influence of all treated fabrics on shear tensile strength and strain against values of pure resin (see Table 2). The significance of the treated fabric is listed in the first half of Table 2. The second half of the table listed the results of non-treated fabric. Comparison of treated and non-treated fabric confirms the positive influence of sodium hydroxide NaOH. Table 2 also directly reports for each fabric the significance value (*p*-value) in comparison with pure resin.

**Figure 6:**
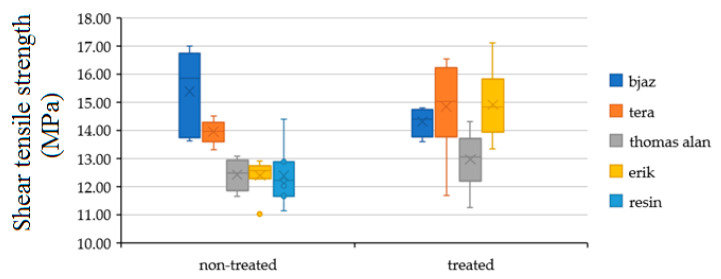
Shear tensile strength results of adhesive bonds with treated and non-treated fabrics by static test.

Figure 6 shows shear tensile strength of the hybrid adhesive bond with treated and non-treated fabric using the static test. The treatment by sodium hydroxide (NaOH) in 5% solution had a positive effect on shear tensile strength of all fabric except fabric Bjaz. The lower strength of treated Bjaz against non-treated was not statistically proved. The low value of the Bajz fabric can be explained by insufficient treatment. The most significant increase of strength against pure resin appeared at the treated fabrics Erik up to 19% on 14.90 ± 1.15 MPa and Tera up to 21% on 15.28 ± 1.05 MPa. 

**Figure 7:**
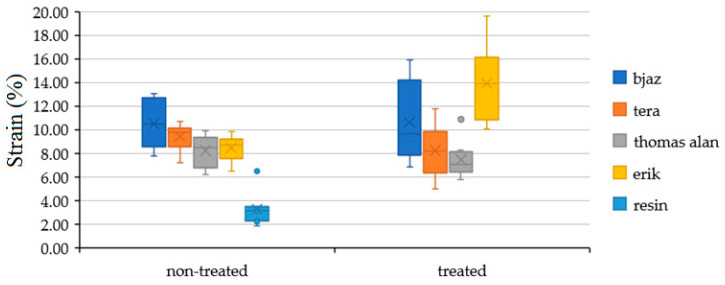
Strain results of adhesive bonds with treated and non-treated fabrics by static test.

Figure 7 shows the strain of the hybrid adhesive bond with treated and non-treated fabric with the static test. All treated fabrics significantly increased the adhesive bond strain, as seen in Table 2. The largest strain increase appeared with the Erik, up 10% to 13.89 ± 3.11%.

**Figure 8:**
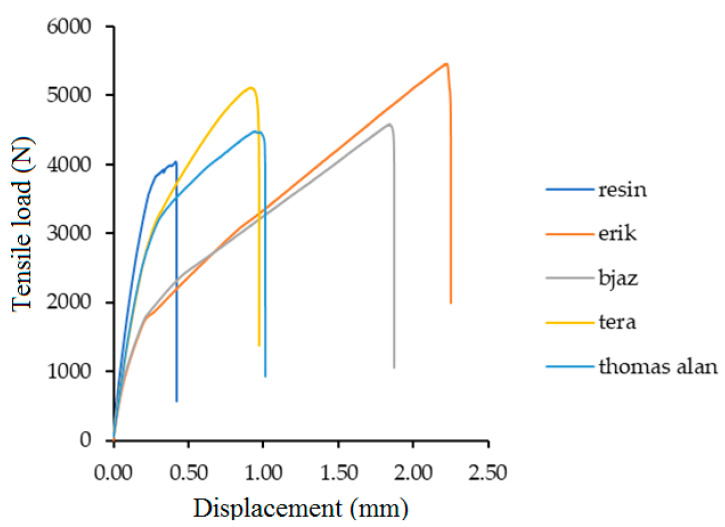
Process of tensile loading vs. displacement of adhesive bonds with treated fabrics in the static tensile test.

Figure 8 shows only the treated fabric, where the tensile load versus displacement is presented. It is obvious that increasing loading force increased displacement of the adhesive bonds with treated fabrics. This dependence of two factors and the change in quasi-static conditions was found. 

The overall results of static tensile tests confirmed sustainability of treatment by sodium hydroxide (NaOH) in 5% solution and the positive effect of fabric treatment on the mechanical properties of the hybrid adhesive bond. For this reason, the fabric treatments were only used following the quasi-static test. The results of the quasi-static tests with lower loading interval 5–50% (192–1951 N) and higher loading interval 5–70% (192–2732 N) are listed in Table 3. The adhesive bonds with simple resin did not resist either quasi-static tests with loading intervals of 5–50% or 5–70% in the required number of finish tests for statistical significance. Table 3 shows the shear tensile strength and strain as well as strain difference in the interval 1–1000 cycles (∆Ɛ), number of finished tests and statistically significant difference (*p*-value) between the same hybrid adhesive bonds in the static test and quasi-static test.

**Figure 9:**
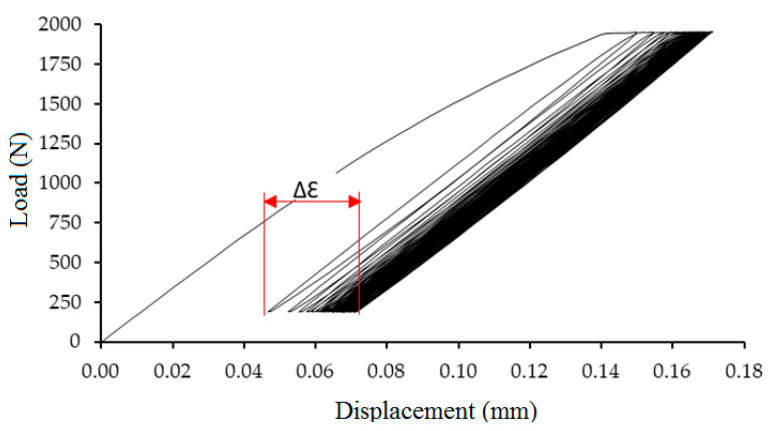
Strain difference in interval 1–1000 cycles (∆Ɛ) at quasi-static loading.

Figure 9 presents quasi-static loading where the strain difference in interval 1–1000 cycles (∆Ɛ) is shown. The strain difference presents a difference between deformation after the 1st cycle and the 1000th cycle. The strain difference is dependent on load and displacement. This value influences the overall course of loading and resulting mechanical properties. The result proved that increasing values of strain difference (∆Ɛ) negatively influenced the strength of the hybrid adhesive bond until bond failure.

**Figure 10:**
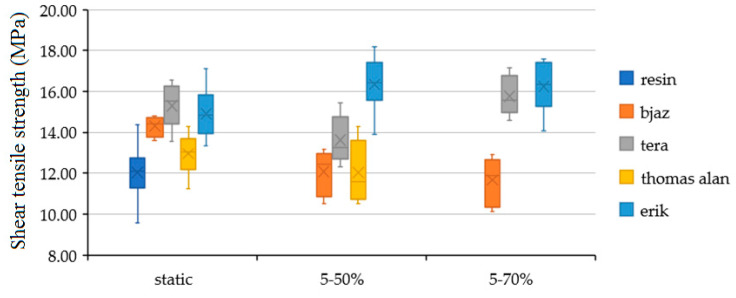
Shear tensile strength with static test, quasi-static test with loading interval 5–50% (192-1951 N) and quasi-static test with loading interval 5–70% (192–2732 N).

Figure 10 shows the results of shear tensile strength during the static test, quasi-static test with loading interval 5–50% (192–1951 N) and quasi-static test with loading interval 5–70% (192–2732 N). The figure shows statistically significant increases of adhesive bond strength with treated fabric at static test; strength values are listed in Table 2. Adhesive bonds with pure resin did not resist quasi-static loading. The strength of adhesive bonds with fabrics decreased at quasi-static test 5–50% except the fabric Erik. The adhesive bonds with the fabric Erik increased against the static value up to 10% to 16.34 ± 1.24 MPa. The adhesive bonds with fabric Thomas Alan did not resist the quasi-static test 5–70%. The strength of adhesive bonds with fabric Bjaz decreased again at loading 5–70%. The strength of adhesive bonds with fabric Erik at loading of 5–70% was 16.22 ± 1.14 MPa, relatively equal to the value from loading of 5–50%. The strength of the adhesive bonds with fabric Tera at loading of 5–70% increased against loading of 5–50% to equal values as static strength. Adhesive bonds with fabric Tera increased against static values up to 3.1% to 15.75 ± 0.85 MPa. The quasi-static test results of strength showed that 100% cotton fabric absorbed cyclic loading with a low decrease of the strength and even with an increase of the strength, i.e., the fabrics Erik and Tera had a self-toughening effect.

**Figure 11:**
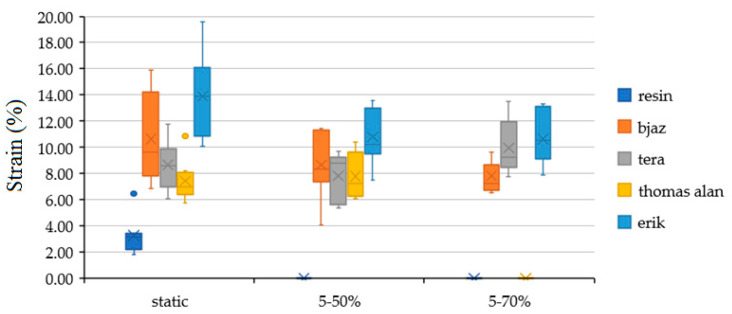
Strain at static test, quasi static test with loading interval 5–50% (192–1951 N) and quasi static test with loading interval 5–70% (192–2732 N).

Figure 11 shows the results of strain with static test, quasi static test with loading interval 5–50% (192–1951 N) and quasi static test with loading interval 5–70% (192–2732 N). Adhesive bond strain with all tested fabrics significantly increased against pure resin with the static test. The most increase with the static test of strain appeared with adhesive with Erik up to 10% to 13.89 ± 3.11%. The fabrics significantly increase strain with the static test. This fact means a good assumption for good mechanical properties with the quasi-static test. The strain of adhesive bonds with the quasi-static test did not significantly change, except for the adhesive bonds with Erik. The strain of adhesive bonds with Erik significantly decreased up to 3% at 5–50% and 5–70% against static values. 

**Figure 12:**
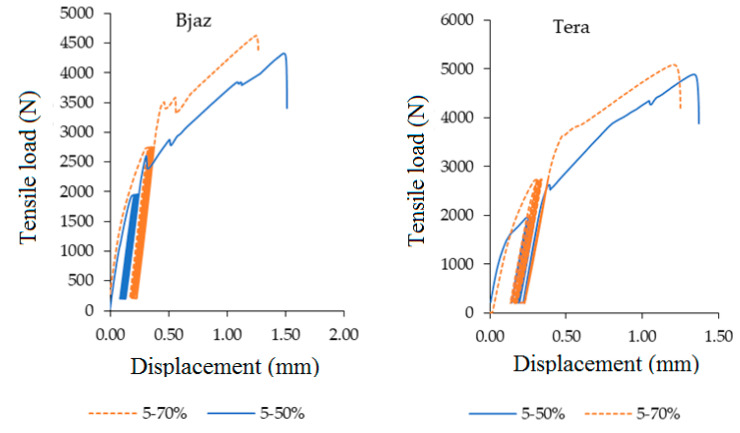
Quasi-static loading process 5–50% and 5–70% for adhesive bonds with fabric Bjaz and Tera.

**Figure 13:**
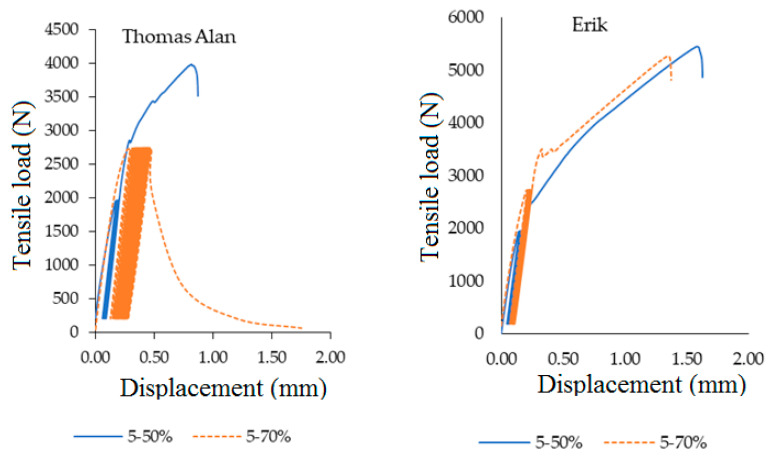
Quasi-static loading process 5–50% and 5–70% for adhesive bonds with fabric Thomas Alan and Erik.

The strain with the quasi static test is very important and can predict future failure. The fabrics increase the strain of the adhesive bond, but the positive effect of the increase has its own limits. Too high a value of deformation can cause a rapid decrease of strength or a failure of the adhesive bond. The strain difference (Figure 9) is an important factor which describes the deformation course. Figure 12 and Figure 13 describe the loading course dependent on tensile load and deformation. Figure 12 describes the loading course of adhesive bond Bjaz with a quasi-static test load of 5–50% with a strain difference of 0.37 ± 0.20% and with the quasi-static test load of 5–70% with a strain difference of 0.43 ± 0.09%. The second part of Figure 12 describes the loading course of adhesive bond Tera at the quasi-static test load of 5–50% with a strain difference of 0.24 ± 0.18% and at a quasi-static test load of 5–70% with a strain difference of 0.35 ± 0.18%. 

Figure 13 describes the loading course of adhesive bond Thomas Alan at the quasi-static test load 5–50% with strain difference 0.19 ± 0.05%. The adhesive bonds did not resist the quasi-static test load 5–70%. A large increase of strain difference at Thomas Alan was evident. The second part of Figure 13 describes the loading course of adhesive bond Erik at quasi-static test load 5–50% with strain difference 0.28 ± 0.16% and at quasi-static test load 5–70% with strain difference 0.36 ± 0.18%.

The results of strain difference at adhesive bonds with Tera and Erik confirmed that the lower value of the difference during cyclic loading positively influenced the following shear tensile strength. The overall results reported the hybrid adhesive bonds with the Erik and Tera fabrics as the most resistance combination. Looking back to Table 1, it is obvious that the density of fabrics is different, but the weft and warp, geometry and layer thickness are similar. The fabric Erik had the best results in all loading conditions with lower density than Tera. On the other hand, the self-toughening process appeared on the fabric Tera, which had the highest density at quasi-static test 5–70%. This means that the right choice of fabric type depends on the condition of use.

## 4. Conclusions

The quasi-static shear performance of hybrid cotton-epoxy composite was studied and the following conclusions are derived:

- SEM analysis proved good wettability of alkali treated cotton fabrics with NaOH solution in a composite layer of hybrid adhesive bonds. Alkali treatment of cotton fabrics also significant influenced mechanical properties.
- Static tests proved the influence of reinforcing cotton fabric in hybrid adhesive bonds. The most significant increase of strength against pure resin appeared at the alkali treated fabric Erik up to 19% on 14.90 ± 1.15 MPa and fabric Tera up to 21% at 15.28 ± 1.05 MPa. The largest strain increase appeared at the Erik, up 10% to 13.89 ± 3.11%.
- The adhesive bonds with simple resin did not resist either quasi-static tests with loading interval 5–50% or 5–70%. Reinforcing cotton fabric resisted both quasi-static tests, except Thomas Alan. Even adhesive bonds with the fabric Erik increased against the static value up to 10% at 16.34 ± 1.24 MPa. The strain at quasi static test is very important and can predict future failure. The fabrics increase the strain of the adhesive bond, but the positive effect of the increase has its own limits. Too high a value of deformation can cause a rapid decrease of strength or failure of the adhesive bond. The results of the strain difference of adhesive bonds with Tera and Erik confirmed that lower values of the difference during cyclic loading have positive influences following shear tensile strength.

## Figures and Tables

**Table 1:** Reinforcing fabric and hybrid adhesive bonded composite details [62].

Fabric	Geometry	Areal Density	Warp Way Strength (200 × 50 mm^2^)	Weft Way Strength (200 × 50 mm^2^)	Composite Layer Thickness
		g·m^−2^	N	N	µm
BJAZ	Plain	145	400	400	530 ± 14
TERA	Plain	305	950	900	312 ± 14
THOMAS ALAN	Twill	230	850	500	342 ± 6
ERIK	Plain	190	850	800	312 ± 14

**Table 2:** Results of static test with treated and non-treated fabrics.

		Static Load Test				*p*-Value	
Hybrid Adhesive Bonds		Shear Tensile Strength		Strain		Shear Tensile Strength	Strain
		MPa		%			
RESIN		12.02	±1.29	3.56	±1.54	-	-
BJAZ	treated	14.30	±0.45	10.62	±3.16	0.03	0.00
TERA		15.28	±1.05	8.66	±1.77	0.00	0.00
THOMAS ALAN		12.95	±0.92	7.44	±1.48	0.03	0.00
ERIK		14.90	±1.15	13.89	±3.11	0.00	0.00
BJAZ	non-treated	15.38	±1.34	10.50	±1.98	0.02	0.09
TERA		13.94	±0.38	9.41	±1.09	0.16	0.19
THOMAS ALAN		12.42	±0.55	8.23	±1.29	0.21	0.30
ERIK		12.39	±0.55	8.43	±1.04	0.00	0.00

**Table 3:** Results of quasi-static test with lower loading interval 5–50% (192–1951 N) and higher loading interval 5–70% (192–2732 N) of adhesive bonds with treated fabrics.

	Adhesive Bond Type		Shear Tensile Strength		Strain		∆Strain (1–1000 cyc.)	Finish Tests	Shear Tensile Strength	Strain
			(MPa)		(%)		(%)	(-)	(*p*-Value)	
Quasi-static 5–50%	RESIN	0		0		0		5/8	-	-
	BJAZ	12.08	±1.00	8.67	±2.41	0.37	±0.20	8/8	0.00	0.11
	TERA	13.61	±1.08	7.83	±1.71	0.24	±0.18	8/8	0.01	0.41
	THOMAS ALAN	12.02	±1.37	7.78	±1.61	0.19	±0.05	8/8	0.18	0.71
	ERIK	16.34	±1.24	10.79	±1.98	0.28	±0.16	8/8	0.04	0.04
Quasi-static 5–70%	RESIN	0		0		0		4/8	-	-
	BJAZ	11.67	±1.01	7.81	±1.08	0.43	±0.09	8/8	0.00	0.02
	TERA	15.75	±0.85	9.94	±1.90	0.35	±0.18	8/8	0.41	0.25
	THOMAS ALAN	0.00	±0.00	0.00	±0.00	0.00	±0.00	4/8	0.00	0.00
	ERIK	16.22	±1.14	10.63	±1.92	0.36	±0.18	8/8	0.06	0.04
